# Acknowledgment to the Reviewers of *Reports* in 2022

**DOI:** 10.3390/reports6010002

**Published:** 2023-01-18

**Authors:** Reports Editorial Office

**Affiliations:** MDPI AG, St. Alban-Anlage 66, 4052 Basel, Switzerland

High-quality academic publishing is built on rigorous peer review. *Reports* was able to uphold its high standards for published papers due to the outstanding efforts of our reviewers. Thanks to the efforts of our reviewers in 2022, the median time to first decision was 20 days and the median time to publication was 39 days. Regardless of whether the articles they examined were ultimately published, the editors would like to express their appreciation and thank the following reviewers for the time and dedication that they have shown *Reports*:
Abdolhassan KazemiKarolina M. StepienAkihiro KanamoriKeisuke HagiharaAldo PezzutoKiran SandhuAlejandro DashtiKonstantinos SpanosAlessandro PoggiKrzysztof LetachowiczAlexandru Florin RogobeteLi LiAlexei ChukhlovinLuigi BennardoAlida Fe TalentoŁukasz A. PoniatowskiÁlvaro Astasio PicadoLyle BurdineÁlvaro MaciasMarcel AdlerAmir Houshang ShemiraniMaria MarinoAna Isabel SaniMario DiplomaticoAncel JulienMaximilian Andreas StorzAndre WirriesMichal OrdakAndreia GeraldoMichela GabelloniAndrew R. MoorheadMichelangelo LucianiAnkit GilaniMing-Tse KuoAnna CzopekMomcilo JankovicAntónio José Madeira NogueiraNadinne RomanBrandon J. BeddingfieldNiels LorenzenCamelia UngureanuNobuto NakanishiChan-Yen KuoOgnjen BarcotChenyu SunPasek JarosławChia-Hung ChouPatrick ButayeChristian BarbatoPengfei ZhangConstantinos TsioutisPetca Razvan-CosminCornelis P. Van De VenPetros IoannouDaniel López MaloPia Lopez-JornetDaniele CorboPierangela TottaDesirée Mena-TudelaRadu-Stefan MiftodeDorin IonescuRaquel Fernandez-GarciaElena FicoRaymond P. GoodrichEmmanouil MagiorkinisRoberto Amerigo PapiniFabrizio D'AcapitoRola JaafarFaris Al-LamiSabine Scholl-BuergiFelix SchmittSeungyup LeeFerruccio ConteSheng-Hung ChenFrancesco SessaShinichi SakamotoFrancisco Fueyo GonzálezSilvia GiovanniniGautam N. ShenoySimone MorraGautam PareekSlaven AbdovićGeorgios SamakovitisSotirios KakavasGian Luigi CanataSubir Kumar JuinGianluca EspositoSvjetlana MohrmannGiovanni TarantinoTadashi FuruyamaGiuseppe MasciaTakuji TanakaGrzegorz TrybekTamas LetohaHaruto NishidaTatsushi MutohHeinz KleinöderTengteng YuHideo KatoTinakon WongpakaranHirotaka NakashimaTomoaki TakataHiu Chuen LokVelasco CimicaHugo CordeiroViorica PatruleaIsao OtsukaVivek PandeyIzabela ZakrockaVivornpun SanprasertJae-Sung YooVlaho BrailoJenny SherriffWalter FlapperJohannes SchulzeXiaxia DiJohn A. St. CyrYajuan SuJose PerdomoYi-Ting HwangJoseph PikkelYuhki SatoJuan Torres-MachoYung Hsiang ChenKaiming BiYutaka MatsudaKaixin LiangZargari Marandi RamtinKamalendra SinghZoran Miloradovic


